# Reversible Humidity Sensitive Clothing for Personal Thermoregulation

**DOI:** 10.1038/srep44208

**Published:** 2017-03-10

**Authors:** Ying Zhong, Fenghua Zhang, Meng Wang, Calvin J. Gardner, Gunwoo Kim, Yanju Liu, Jinsong Leng, Sungho Jin, Renkun Chen

**Affiliations:** 1 Materials Science and Engineering Program, University of California, San Diego, La Jolla, CA 92093, USA; 2 State Key Laboratory of Advanced Welding and Joining, Harbin Institute of Technology, Harbin 150001, China; 3 Center for Composite Materials and Structures, Harbin Institute of Technology, Harbin 150080, China; 4 Department of Structural Engineering, University of California, San Diego, La Jolla, CA 92093, USA; 5 Department of Astronautical Science and Mechanics, Harbin Institute of Technology, Harbin 150001, China; 6 Department of Mechanical and Aerospace Engineering, University of California, San Diego, La Jolla, CA 92093, USA

## Abstract

Two kinds of humidity-induced, bendable smart clothing have been designed to reversibly adapt their thermal insulation functionality. The first design mimics the pores in human skin, in which pre-cut flaps open to produce pores in Nafion sheets when humidity increases, as might occur during human sweating thus permitting air flow and reducing both the humidity level and the apparent temperature. Like the smart human sweating pores, the flaps can close automatically after the perspiration to keep the wearer warm. The second design involves thickness adjustable clothes by inserting the bent polymer sheets between two fabrics. As the humidity increases, the sheets become thinner, thus reducing the gap between the two fabrics to reduce the thermal insulation. The insulation layer can recover its original thickness upon humidity reduction to restore its warmth-preservation function. Such humidity sensitive smart polymer materials can be utilized to adjust personal comfort, and be effective in reducing energy consumption for building heating or cooling with numerous smart design.

Around 48% of the building energy consumption in the United States is attributed to temperature control using heating, ventilation and air conditioning (HVAC) systems^1^,^2^,^3^,^4^. If the average set-points of the indoor temperature are extended by a few degrees in either direction, the energy consumption in building HVAC systems will be substantially reduced^5^,^6^. Therefore, instead of controlling the environment of the entire building, it is highly desirable to reduce the overall energy consumption via personal thermoregulation^7^,^8^.

Toward this end, there is a tremendous effort to develop smart clothes which can automatically adapt to ambient temperatures, for example, using wearable electronic textiles^9^,^10^,^11^. Smart thermally adaptive textiles with passive thermoregulating mechanisms may allow for simpler textile constructions and improved energy efficiency by taking advantage of the essential thermal effect of materials such as temperature-sensitive shape memory polymers (SMPs)^12^,^13^. However, SMPs in general exhibit a one-way shape memory effect at certain temperatures which cannot reversibly adapt to continuous fluctuations of the external environment. Though some materials are capable of two-way shape memory behavior, thus far their sensitivity to small variations in temperature is too low to accommodate the thermoregulatory demands of the human body^14^,^15^,^16^. Moreover, the sensitive and effective temperature ranges of sensors and materials are fixed parameters^17^,^18^, yet the comfort of human bodies may be affected by wide ranging factors such as gender, health, or age, and individuals may feel differently even at the same temperature^14^,^19^. Therefore, it is imperative to develop systems which can reversibly respond to the immediate sensations of the wearer to be used to produce workable smart thermoregulating textiles^20^.

In contrast to the insufficient temperature-based response of SMPs, a humidity-based shape change, which might be activated by human sweat, can be substantially more effective^21^,^22^,^23^. For example, The Nafion polymer from DuPont is a thermo-moisture responsive polymer which can efficiently and reversibly absorb water and swell, with its swelling ratio increasing with temperature at the same absolute humidity^24^,^25^,^26^. As we observed, when subjected to a humidity difference between the opposing faces, a Nafion film will bend towards the lower humidity side in less than one second. The film can then quickly recover the original form once the humidity difference vanishes^27^,^28^. The humidity sensitivity is attributed to the polymeric chains in Nafion including both hydrophobic polytetrafluorethylene backbone and hydrophilic perfluoroether sulphonic acid side chains (-SO_3_H). Upon humidity increase, Nafion can rapidly exchange H^+^ with water and formed a large number of water transport channels, leading to microphase separation between the hydrophobic back bone and the hydrophilic side chain groups and consequently the swelling^24^,^29^,^30^,^31^. By utilizing the humidity responsiveness of Nafion, we designed and fabricated two kinds of smart textile structures: the first mimics the sweat pores in human skin and the second responsively varies in thickness to adjust clothing thermal insulation. By using these two designs, we are expecting to extend the temperature adaptability of human skins and reduce the energy consumption of HVAC systems.

## Results

### Human sweat pore mimicking design

**Figure 1:**
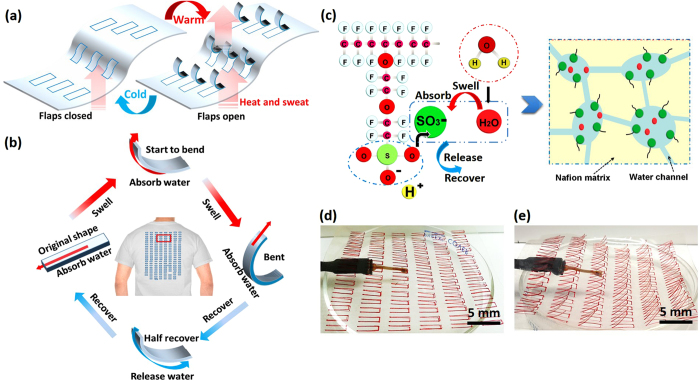
(**a**) Nafion sheet schematics with openable flaps mimicking thermo-adaptive functionality of human skin; (**b**) reversible swelling, bending, release and recovery behavior of one single Nafion flap; (**c**) swelling and water transport mechanism of Nafion; (**d**) Nafion sheet with closed flaps, underneath is a humidity/temperature sensor; (**d**) Nafion sheet with opened flaps when placed upon water vapor above a be placed near sweating environment. After the film was moved away from the water bath and its associated vapor, the flaps closed within seconds as well. This implies that, once the heat and simulated sweat were to be released, equilibrium of humidity would be restored, and the expansion of both the interior and exterior sides of the Nafion flaps equalized, which relieved the built-up stresses and closed the flaps automatically (**d**) to restore the function as a typical insulative cloth (left turning part of **a,b**). This cyclic process is repeatable, fast, and sensitive for many cycles without degradation in functionality, enabling an individually sensitive smart textile actuated by the wearer’s physical comfort without requiring any external power.

To mimic human sweat pore functionality, Nafion films with flaps are designed as shown in Fig. 1a,b. Upon temperature increase, a wearer will sweat and build up a higher humidity on the inner surface of the textile, i.e., the lower side facing the skin or a vapor chamber. As seen in the upper part of Fig. 1a,b, the inner sides of the Nafion flaps swell upon water uptake. As shown in Fig. 1c, the increased humidity causes the microphase separation between the hydrophobic bone (C-F) and hydrophilic side chain groups (SO_3_-). Nafion can easily exchange the H^+^ with water and form numerous water transport channels, leading to rapid swell to achieve the equilibrium. As the outer surface is exposed to the ambient environment with a lower humidity, the inner side of the Nafion flap swells more than the outer portion, creating a differential expansion that causes the flaps to bend upward and open up the pores. The steepness of the flex increases with the magnitude of the humidity gradient across the film, and is bounded by the maximal absorption and expansion levels of the material. Figure 1d,e show the response of the Nafion flaps without and with water vapor (32 °C, 90% RH), respectively. A quick dynamic cycling procedure is also shown in Movie S1: upon contacting the water vapor from a warm water bath, the Nafion flaps opened within a few seconds. This quick pore opening response could allow for prompt sweat evaporation and heat release. The samples were also kept at a constant high humidity (around 90% RH) throughout the test and the open status of the flaps was found to be preserved during the 5-hour duration. It should be noted that Nafion is highly permeable to water, so the humidity gradient across the membrane will gradually decrease over time, but will not vanish because of the humidity difference between “skin” and the ambient. Therefore, we chose a relatively thick Nafion (0.183 mm) to achieve the long-term flap opening status. Further optimization of the Nafion thickness and other design parameters (such as shapes and sizes of the flaps) would warrant future investigation.

**Figure 2:**
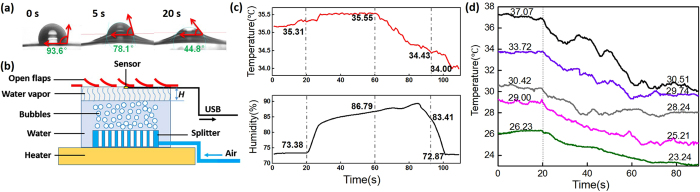
(**a**) Contact angel and water up-taken swelling of Nafion film at 0 s, 5 s, and 20 s; (**b**) schematic of a sweat simulation system; (**c**) humidity and temperature response of the “sweating” skin simulation system working at 35 °C; (**d**) temperature response upon flap opening at various working temperatures (dotted line separates flap close and open zone).

The wetting and swelling behavior of Nafion film with a droplet of water on it is shown in Fig. 2a. Upon touching, the contact angle was 93.6°. After 5 s, the contact angle reduced to 78.1°, and the area underneath the water droplet hunched because of swelling; in the meanwhile, the volume of the water droplet shrank by 50%. After 20 s, the contact angle became as low as 44.8° with more upheaval of the film and more volume shrink of the water droplet. This process is a clear evidence for the rapid water up-taken and swelling behavior of the Nafion film. A system as shown in Fig. 2b is employed to mimic the sweating of human body and study the thermo-adaptive function of Nafion at different temperatures. By controlling the air flow rate as well as the distance between the Nafion sheet and the water bath surface, both the humidity and the temperature were regulated at the inner surface of the Nafion sheet. Figure 2c is mimicking the humidity and temperature response of sweating human skin covered by this design. The “sweating” began, meaning the humidity increased rapidly, at the twentieth second. After 40 s, the humidity reached 86.8% and triggered the flaps to open. The temperature started to decrease because of the flaps, and the temperature reduction could reach 1.1 °C until the flaps closed at a humidity of 83.4%. The later decrease and stabilization of humidity and temperature are due to the completion of the “sweating”. The heat/humidity release by the automatic opening of the “pores” can lead to a temperature decrease of up to 1.55 °C, which can create a significant cool feeling for human body. The data presented in Fig. 2d display consistent temperature decrease on the inner surface upon flap opening at various working temperatures from 37 °C to 23 °C, with larger cooling effect observed for higher starting temperatures. This observation is consistent with our initial expectation that a higher temperature and humidity gradient results in faster response and larger swelling ratios, which lead to higher air flow and faster evaporative cooling. As opposed to other temperature-responsive materials, Nafion reacts to both temperature and humidity and can assist in thermoregulatory cooling of a body based on the wearer’s own internal regulation system of perspiration, and is less dependent on the environmental temperature. Moreover, the effective temperature reduction occurs within 1 min (e.g. 1 min for 6.56 °C reduction from 37.07 °C as shown in Fig. 2d), which is much faster and more efficient than most HVAC systems which have to cool down the entire surrounding environment. With this smart sweating pore mimetic structure, one can extend the HVAC setpoints to a higher temperature, e.g., from 26 °C to 29 °C.

### Thickness-changeable design

**Figure 3:**
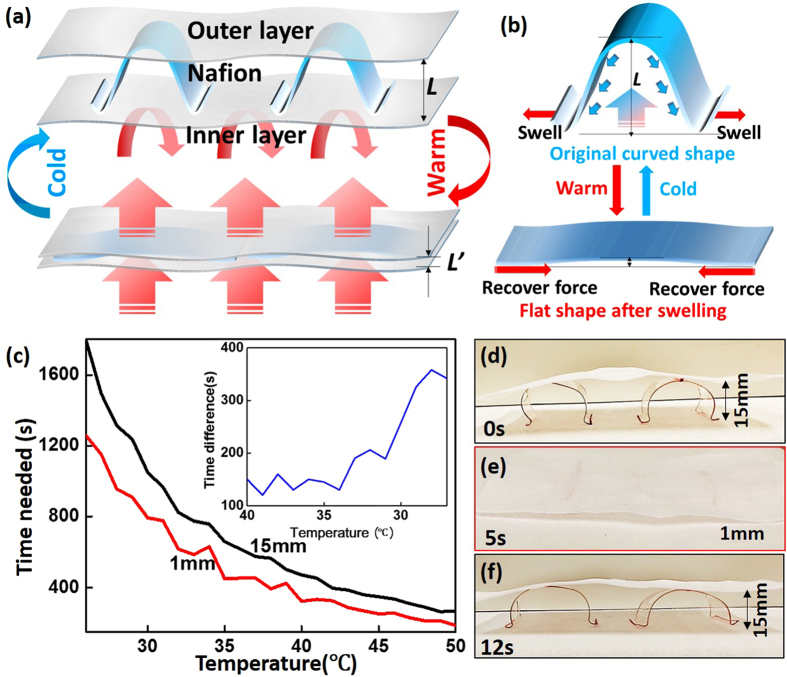
(**a**) The schematic of a smart, thickness reversible structure using Nafion as a thermally adaptive interlayer; (**b**) upper: one arched Nafion ribbon flattens and reduces the thickness of the insert layer while humidity-swelled; lower: Nafion ribbon flexes and recovers the original arch status to offer good insulation with thicker insert layer; (**c**) the time needed to cool down one degree Celsius with different insulation thicknesses, with the time difference vs. temperature shown in the inset graph; (**d**) photograph of the arched Nafion ribbon interlayer; (**e**) after 5 seconds upon exposure to increased water vapor (e.g., by placing the composite fabric structure above a warm water bath), the vaulted Nafion sheet stretched to a near-flat geometry with the outer nylon layer almost in contact with the inner layer; (**f**) after moving away from the water vapor for 7 seconds, the Nafion interlayer recovered to its original arch shape and restored the air gap to the original thickness.

Figure 3 shows a possible design for a passively activated thickness-adjustable textile using a Nafion-based smart interlayer for adaptive insulation. By constructing a multilayered textile, clothing utilizes the low thermal conductivity of air to prevent heat loss at lower temperature. As seen in the upper part of Fig. 3a,b, Nafion film with an arched shape maintains the separation between the upper and lower layers as thick insulation to prevent heat loss. The highest points of the arched Nafion ribbons were anchored to the outer textile layer and the lowest points were allowed to move freely along the surface of the inner textile layer as the Nafion responded to humidity. If the humidity increases such as caused by warm water bath or wearer’s sweating, the bottom sides of the Nafion ribbons will swell. However, as the humidity at the top side of the Nafion ribbon is much lower, leading to much less extension in length. Therefore, there is an internal stress imbalance generated between the longer bottom side and the shorter top side. As the two low contact points have no constraint along the length direction, they would like to slide towards opposite directions to flatten the Nafion ribbons and release the internal stress, which ends up with decreasing the interlayer thickness as shown in the lower part of Fig. 3a,b. Once the wearer stops sweating, the humidity gradient diminishes and the ribbons recover their arched shape within seconds and restore the original thicker dimension status. The real time insulation effect of the thicker and flexed (15 mm air gap) versus thinner and flatter (1 mm air gap) interlayer was tested and shown in Fig. 3c. The time needed to reduce the temperature by one degree Celsius for the thicker interlayer was hundreds of seconds longer than the thinner interlayer, indicating that thinning the insulation layer can accelerate heat and humidity diffusion, and vice versa. This time difference (Fig. 3c inset) is longer at lower temperatures, meaning this thickness adjustment design can be extremely beneficial in cooler environment. The prompt response time can be seen in Movie S2 and in Fig. 3d–f. Upon exposure to a humidity gradient, the arched ribbons supporting an interlayer thickness of 15 mm flattened within 5 seconds. After removing the humidity gradient, the Nafion ribbon fully recovered to its arch form in 7 seconds. As shown in Movie S2, this repetitive procedure remains fast and reversible for many cycles without degradation. Since this procedure is reversible, the smart clothing can be designed to keep the user warm by default, e.g., in winter, and then to automatically aid the body to cool down when the wearer sweats. The thickness adjustable design enables the use of only one garment for a much wider temperature window and could reduce the energy consumption of building HVAC, especially in cold weather.

### Recycle and repeatable use

**Figure 4:**
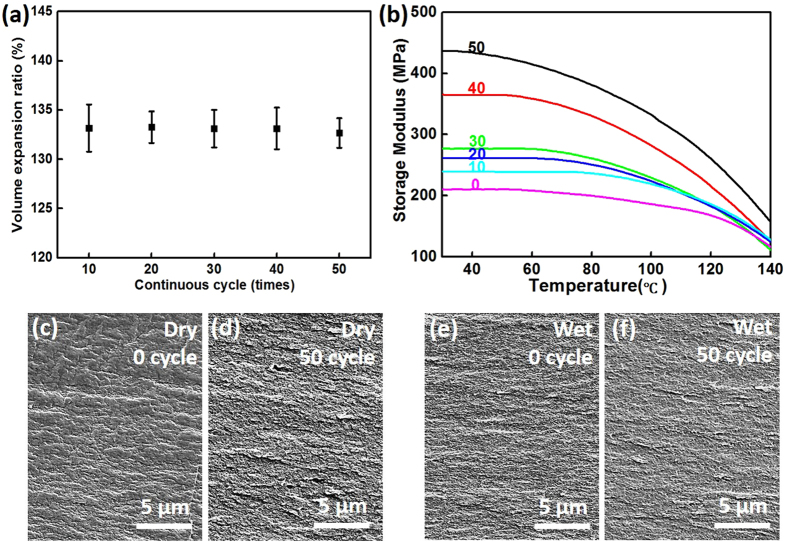
(**a**) The volume expansion ratio and (**b**) storage modulus of Nafion film after several tens cycles of times of water swelling. The number above each curve indicates the cycle numbers; (**c**) the cross section of dry Nafion (0 cycle) after tensile test; (**d**) the cross section of dry Nafion (50 cycles) after tensile test; (**e**) the cross section of Nafion after water uptake (0 cycle) after tensile test; (**f**) the cross section of Nafion after water uptake (50 cycles) after tensile test.

Nafion-based humidity sensitive sheets are washable and possess long-term usability and reversibility. Figure 4a shows the volume expansion ratio of Nafion after each 10 swelling cycles in water. It can be seen that after 50 cycles, the volume swelling ratio of the film remained constant at a ratio of around 133% without degradation. The storage modulus of Nafion film was also investigated after various water swelling cycle numbers using dynamic mechanical analyzer (DMA). According to Fig. 4b, the storage modulus of Nafion increases with the number of cycles. This indicates that the strength of the film can be enhanced by water swelling cycles, especially within the temperature range for smart clothing applications, possibly benefiting the stability of the open flaps in the human sweat pore mimic design as well as the arched status to support the upper layer in the thickness adjusting design. In Fig. 4c–f, we compared the fracture surface of Nafion films after tensile tests under different conditions (same tensile speed): dry before water absorption, dry after 50 cycles of water absorption, wet before water absorption. It turned out that films under wet condition and experienced more cycle times tend to show denser fracture micro-structure, meaning longer path and more energy consumption during the crack propagation, which can explain the enhanced storage modulus. For the open and close cycles, the maximum cycles conducted are 400 times. It still maintains the switching property indicating excellent durability and repeatability.

## Discussion

**Figure 5:**
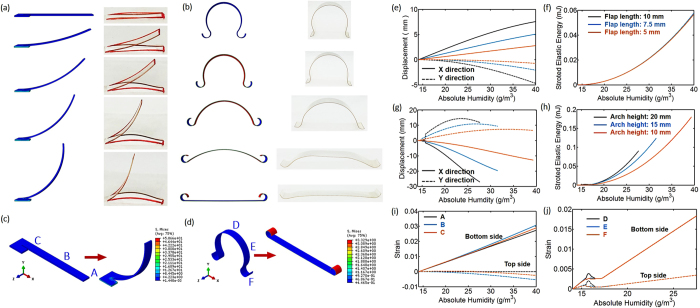
(**a**) Comparison of deformation between experimental observation and numerical simulation – deformation contour of flap structures with bottom absolute humidity of 14.0, 20.5, 27.0, 33.5, and 40.0 g/m^3^ (from top to bottom); (**b**) Comparison of deformation between experimental observation and numerical simulation – deformation contour of arch structures with bottom absolute humidity of 14.0, 17.5, 21.0, 24.5, and 27.5 g/m^3^ (from top to bottom); (**c**) stress distribution in deformed 10-mm-long flap structure at bottom absolute humidity of 40.0 g/m^3^; (**d**) stress distribution in deformed 25-mm-high arch structure at bottom absolute humidity of 27.5 g/m^3^; (**e**) strain development with absolute humidity on bottom and top side of Node A, B, C in flap structure; (**f**) Node A displacement along X and Y coordinate with flap length of 10 mm, 7.5 mm, and 5 mm as a function of absolute humidity at structure bottom; (**g**) store elastic energy in deformed flap structure with flap lengths of 10 mm, 7.5 mm, and 5 mm as a function of absolute humidity at structure bottom; (**h**) strain development with absolute humidity on both bottom and top side of arch structure (arch height 10 mm); (**i**) Node D displacement along Y coordinate, and Node F displacement along X coordinate with arch height of 20 mm, 15 mm, and 10 mm as a function of absolute humidity at structure bottom; (**j**) store elastic energy in deformed arch structure with arch height of 20 mm, 15 mm, and 10 mm as a function of absolute humidity at structure bottom.

To understand the mechanical response of above designs, finite element analysis (FEA) was conducted by using the ABAQUS standard package. Figure 5a,b show the simulated deformation contours of flap and arch structures agree well with the experimental observation at different humidity levels. The photos of the samples were taken after the humidity reached the same value (14.0, 20.5, 27.0, 33.5, and 40.0 g/m^3^) as used in the simulation. The deformation contours of the flap (like open angle, curvature, etc.) and the arch (like height, length, curvature, etc.) matched very well with the simulation. As shown in Fig. 5a,c, the flap structure deforming upwards with the increase of the humidity from the bottom. The majority of stress concentrates in the root region, but the stress level in the body of the flap is relatively low. Figure 5e is the strain distribution on the bottom side and top side of node A, B, C in Fig. 5c under different humidity. Bottom side undergoes tensile strain, while top side experiences compression strain, and both of them increase with humidity (up to 3% on top and −0.5% on bottom side). The middle part of the flap, represented by node B also shows larger strain than the other parts. This distribution of strain is the mean reason for the deformation and open behavior of the flaps. Figure 5f shows the flap tip (node A) displacements in X and Y directions with the same flap thickness but different lengths of 10 mm, 7.5 mm, and 5 mm, representing different length-thickness ratios. Results are showing similar rotation angles under the same humidity, but more displacements for larger length-thickness ratio (as much as 7.5 mm in Y direction for 10 mm model). Stored elastic energy for all the three cases is almost the same under certain humidity. Therefore, as there is no decrease in rotation angle or stored elastic energy with larger length-thickness ratio, we can increase the length of the flap as much as the cloth design allows to increase the flap area for more and faster heat/humidity release.

As shown in Fig. 5b,d, the arch structure deformed downwards with the increasing of humidity at the bottom. The stress (see Fig. 5d) and strain (see Fig. 5h, node D, E, F possess coincident trend) distribution in the arch is very uniform. That is because the arch structure could freely deform outwards with no constrain in horizontal directions. Unlike the flap structure, tensile strain was developed on both sides of the arch structure (see Fig. 5h). The moisture gradient between top and bottom sides results in different strain distributions along the film thickness direction. A fully flattened arch structure has 1.8% of tensile strain on bottom and 0.4% of tensile strain on top. As the majority of moisture was taken by bottom side, expansion would dominant on bottom side, resulting in an increase of tensile strain. Inconsistency in deformation on top and bottom would lead to the curving of flap structure or flattening of the arch structure. Figure 5i shows the displacement of node D and F with different arch heights: 20 mm, 15 mm, and 10 mm. Node F would slide outwards and node D would move downwards with humidity increasing. For 20 mm size arch, a humidity of 27.5 g/m^3^ can fully flatten the arch (meaning the Y direction displacement equals the arch height), but for 10 mm sized arch, the needed humidity to fully flatten the arch is doubled. This can also attribute to the store elastic energy curve shown in Fig. 5i. Therefore, higher arch leads to faster response, which is beneficial for broadening the functional temperature range for this thickness adjustable design.

## Conclusion

In summary, we have designed two kinds of Nafion-based smart clothing structures triggered by humidity change, which can quickly and reversibly change its porosity or thermal insulation in response to an individual’s perspiration level. A sweating pore mimicking design consisting of a flap array structure on a Nafion sheet has been shown to respond to a humidity gradient and automatically opens or closes to regulate air flow through the pores, thereby achieving humidity and temperature control. A double layered, thickness-changeable structure with Nafion ribbons as an insert was also designed and demonstrated to adjust the air gap and alter the thermal insulation between two fabric layers. Both designs are sensitive to humidity and temperature changes and have been successfully demonstrated for reversible operations over numerous cycles. These human skin-mimicking structures work in an adaptive and repeatable manner. If designed into textile geometry, these humidity sensitive polymer structures will extend a wearer’s thermal comfort range and can contribute to energy saving of building HVAC systems.

## Methods

### Materials

Nafion ® N117 with the thickness of 0.183 mm was purchased from DuPont. The specimens were dried and annealed at 130 °C before using.

### Sample preparation

A rectangular array of elongated 2 mm × 10 mm flaps was cut into the film (100 mm × 100 mm) via razor blade with 4 mm horizontal pitch and 15 mm vertical pitch. (2) Nafion ribbons (10 mm × 40 mm) for the thickness adjustment design were molded into an arched shape with a height of 15 mm at temperature higher than its glass transition temperature (130 °C).

### Humidity and temperature measurement

The humidity and temperature measurements were carried out using a digital humidity sensor SHTC1 (RH/T) (Sensirion Co. Ltd.), which can measure humidity and temperature simultaneously. The accuracy tolerance for its relative humidity and temperature measurement is ± 3%RH and ± 0.3 °C, respectively. The resolution is 0.01%RH and 0.01 °C, respectively. The dimension of the sensor is 2 × 2 × 0.75 mm^3^, which is one of the smallest commercialized humidity sensors to guarantee the accuracy of the targeted location.

### Angle measurement

All the angle measurements were done by taking pictures with a camera with its lens positioning perpendicular to the substrate or the film. The pictures were then loaded into a photography software to draw a tangent line and measure its angle with the substrate or the original film. For each reported data point, the pictures were collected from at least five samples, and each picture was measured 5 times to get the average value to minimize the measurement error.

### Water Uptake Performance

The Nafion film was cut into 20 mm × 20 mm squares and immersed into water for 3 min and then dried at 70 °C repeatedly. The volume expansion ratio was obtained by measuring the new length, width and thickness of the film every 10 cycles and divide their product with the original one.

### Dynamic Mechanical Analysis (DMA)

The dynamic thermo-mechanical properties of Nafion film were carried out using a dynamic mechanical analyzer (DMA, TA Q800) with a heating rate of 10 °C/min and frequency of 2 Hz. The dimensions of the samples were 5.5 mm × 2.8 mm. Different samples were measured for different cycle times.

### Mechanical Response Finite Element Analysis (FEA)

Mechanical response finite element analysis (FEA) was conducted by using ABAQUS standard package. To replicate the confinement of film on the flap, one unit of the flap was modeled with 2 mm wide fringes at the root. All boundary displacements were fixed to zero at the root of the flap. In order to save computational cost, the arch structure was modeled to be 2-mm-wide half structure, with both symmetric boundary conditions along width directions and at the middle span of the arch. The bottom of the arch was set to be rolling-free in the horizontal direction. For the flap structure, displacement in X and Y direction were recorded at node A; for the arch structure, the displacement in X direction was recorded at F, and Y direction was recorded at D. For both designs, the strain was recorded at three different locations, on both top and bottom sides of the structure as A, B, C and D, E, F respectively for each design.

Moisture boundary condition was applied at both top and bottom sides of these two structures. On top of the structure. An ambient humidity was applied, with 70% relative humidity at 23 °C, which is equivalent to an absolute humidity of 14 g/m^3^. The humidity of vapor was linearly increased on the bottom of the structure until reaching 92% relative humidity at 37 °C, which is equivalent to an absolute humidity 40 g/m^3^. According to Fick’s law, under steady state, a linear distribution of moisture along film thickness direction was assumed.

Three different cases were calculated, with the length or height of model to be 100%, 75 and 50% of the original size. Linear-elastic model was adopted in the simulation. The elastic modulus and Poisson’s ratio were taken to be 275 MPa and 0.487, respectively. The isotropic uniaxial expansion ratio was set to be 0.002/(g/m^3^). According to mesh sensitivity analysis, the computational results converged when mesh size reduced to a size of 50 mm, leading to four layers of mesh along the thickness direction.

## Additional Information

**How to cite this article:** Zhong, Y. *et al*. Reversible Humidity Sensitive Clothing for Personal Thermoregulation. *Sci. Rep.* **7**, 44208; doi: 10.1038/srep44208 (2017).

**Publisher's note:** Springer Nature remains neutral with regard to jurisdictional claims in published maps and institutional affiliations.
